# With appreciation

**DOI:** 10.1002/ehf2.13721

**Published:** 2021-11-18

We thank the following individuals who served as manuscript reviewers for *ESC Heart Failure* Journal in 2021.

We highly appreciate their efforts.

Fair, conscientious, and timely peer reviews contribute to the success of *ESC Heart Failure*.

The Editors.

## 15 and more reviews

Cristiano Amarelli

Erdi Babayigit

Constantinos Bakogiannis

David Baran

Jan Benes

Francesco Bianco

Antonio Cannata

Maryam Chenaghlou

Hiroaki Hiraiwa

Angelos Rigopoulos

Nicholas Wettersten

## 5–14 reviews

Mahdi Abdollahi‐Karizno

Manyoo Agarwal

Constantina Aggeli

Alberto Aimo

Khawaja Akhtar

Amit Alam

Stefano Albani

Giuseppe Ambrosio

Pietro Ameri

Ahmad Amin

Eisuke Amiya

Øyvind Senstad Andersen

George Chukwuemeka Anene‐Nzelu

Elena Angelopoulou

Mohammad Mostafa

Kazutaka Aonna

Sara Ariotti

Gani Bajraktari

Luca Baldetti

Viktor Bánhegyi

Vincenzo Barbera

Tarek Bekfani

Michael Berchtold‐Herz

Benedikt Bernhard

Michael Böhm

Josip Andelo Borovac

Tomaso Bottio

Kadir Caliskan

Miguel Camafort‐Babkowski

Albino Carrizzo

Stefano Carugo

Vincenzo Castiglione

Elizabeth Cestario

Ovidiu Chioncel

Vijay Chopra

Song‐Yun Chu

Michele Ciccarelli

Giovanni Cimmino

Otavio Coelho‐Filho

Stefano Coiro

Augustin Coisne

Paolo Colombo

Pascal De Groote

Michael Diamant

Konstantinos Dimopoulos

Torsten Doenst

Erwan Donal

Marcus Dörr

Howard Eisen

Mehmet Eren

Iacopo Fabiani

Mauro Feola

Michel Galinier

Elena Galli

Edit Gara

Alberto Giannoni

Nicolas Girerd

Monika Gladka

Rodrigo Gopar‐Nieto

Joshua M. Hare

Toru Hashimoto

Jaime Hernandez Montfort

Clara Hjalmarsson

Stephan Hohmann

Jonathan Howlett

Massimo Iacoviello

David Ishizawar

Noriaki Iwahashi

Amela Juisc

Marwan Jumean

Peter Kennel

Kalliopi Keramida

Attila Kiss

Mitja Lainscak

Ekaterini Lambrinou

Michal Laufer Perl

Daniel Lavall

Yixiu Liang

Gerard Linssen

Goran Loncar

Alessandro Lupi

Josep Lupón

Alexander Maass

Micha Maeder

Hirsaki Makimoto

David Marono

Gurbet Mert

Robert Miller

Marzieh Mirtajaddini

Luca Monzo

Daniel Morris

Dimitrios Mouselimis

Joseph Muhlestein

Jochen Müller‐Ehmsen

Nasim Naderi

Yuichi Notomi

Michel Noutsias

Marish Oerlemans

Alberto Palazzuoli

Vittorio Palmieri

Vasileios Panis

Ernesto Paoletti

Maria Papathanasiou

Maria Concetta Pastore

Dimitri Patriki

Simone Persampieri

Emanuele Pivetta

Eftychia Polyzogopoulou

Ahmad Pratama

Goncalo Proenca

Rasmus Rivinius

Emma Robinson

Francois Roubille

Vanessa Rubesch‐Kütemeyer

Nikolay Runev

Andrea Salzano

Marcus Sandri

Gianluigi Savarese

Nadja Scherbakov

Gabriele Schiattarella

Benedikt Schrage

Hadi Skouri

Jayakumar Sreenivasan

Stephan Stöbe

Carlo Tocchetti

Vassil Traykov

Filippos Triposkiadis

Anastasios Tsarouchas

Francisco Vasques‐Nóvoa

Carmine Dario Vizza

Iosif Xenogiannis

